# Dataset of TWIST1-regulated genes in the cranial mesoderm and a transcriptome comparison of cranial mesoderm and cranial neural crest

**DOI:** 10.1016/j.dib.2016.09.001

**Published:** 2016-09-12

**Authors:** Heidi Bildsoe, Xiaochen Fan, Emilie E. Wilkie, Ator Ashoti, Vanessa J. Jones, Melinda Power, Jing Qin, Junwen Wang, Patrick P.L. Tam, David A.F. Loebel

**Affiliations:** a Embryology Unit, Children’s Medical Research Institute, Sydney Medical School, University of Sydney, Westmead, NSW 2145, Australia; b Bioinformatics Group, Children’s Medical Research Institute, Sydney Medical School, University of Sydney, Westmead, NSW 2145, Australia; c Biomedical Sciences, Faculty of Mathematics and Natural Sciences, University of Groningen, The Netherlands; d Centre for Genomic Sciences, Department of Biochemistry, Li Ka Shing Faculty of Medicine, University of Hong Kong, Hong Kong SAR, China

## Abstract

This article contains data related to the research article entitled “Transcriptional targets of TWIST1 in the cranial mesoderm regulate cell-matrix interactions and mesenchyme maintenance” by Bildsoe et al. (2016) [1]. The data presented here are derived from: (1) a microarray-based comparison of sorted cranial mesoderm (CM) and cranial neural crest (CNC) cells from E9.5 mouse embryos; (2) comparisons of transcription profiles of head tissues from mouse embryos with a CM-specific loss-of-function of *Twist1* and control mouse embryos collected at E8.5 and E9.5; (3) ChIP-seq using a TWIST1-specific monoclonal antibody with chromatin extracts from TWIST1-expressing MDCK cells, a model for a TWIST1-dependent mesenchymal state.

**Specifications Table**

**Table:** 

Subject area	Biology
More specific subject area	Developmental Biology
Type of data	Tables
How data was acquired	Illumina Mouse WG-6 v2 arrays;
	Chromatin-immunoprecipitation and next generation sequencing
Data format	Analyzed
Experimental factors	Samples for microarray analysis were collected from either FACS sorted GFP-positive embryonic head tissues, or whole embryo heads. Chromatin for ChIP-seq was collected from MDCK cells over-expressing human *TWIST1.*
Experimental features	Transcriptome comparison between sorted E9.5 cranial mesoderm (CM) and neural crest cells. *Twist1* conditional knockout and control tissues (E8.5 & E9.5). TWIST1 genomic binding sites in MDCK cells.
Data source location	Children׳s Medical Research Institute, Sydney Medical School, University of Sydney, Australia
Data accessibility	The microarray and ChIP-sequencing data within this article are accessible in GEO under accession number GEO: GSE80663. http://www.ncbi.nlm.nih.gov/geo/query/acc.cgi?acc=GSE80663

**Value of the data**

• The data set provides an important reference for all studies investigating *Twist1* function in the context of development and cancer.
• By comparing the transcriptome of the cranial mesoderm and cranial neural crest, the data set provide a useful tool for studying the complex process of craniofacial development.
• The data set potentially contributes to the identification of genes that control the mesenchymal cell state in development and cancer.

## 1 Data

Dissociated craniofacial tissues that were FACS-sorted by GFP expression reporting either *Mesp1*-Cre or *Wnt1*-Cre activity were compared using microarrays (Supplementary Tables 1 and 2). Dissected embryo heads of control (*Twist1*^flox/+^), heterozygote (Twist1^del/+^), mesoderm heterozgote (*Twist1*^flox/+^*; Mesp1*^Cre/+^) and conditional knockout (*Twist1*^flox/del^*; Mesp1*^Cre/+^) (Supplementary Tables 3 and 4) genotypes were compared using microarrays. Chromatin immunoprecipitation using an anti-TWIST1 antibody was performed on MDCK cells that express human *TWIST1* (Supplementary Table 5).

## 2 Experimental design, materials and methods

### 2.1 Isolation and analysis of CM and CNC populations

Embryo were collected at E9.5 from *Mesp1*-Cre x Z/EG (for CM) and *Wnt1*-Cre x Z/EG (for CNC) [2], [3], [4]. Heads were dissected below the first branchial arch, dissociated and prepared for cell sorting as described [2]. Each sample yielded 4000–18,000 GFP-positive cells, which were stored at −80 °C. RNA was extracted and amplified using Illumina TotalPrep (Ambion) and labeled using MessageAmp II aRNA (Ambion) as described elsewhere [1].

### 2.2 Transcriptomic analysis of *Twist1*-conditional mutant embryos

Embryos in which *Twist1* was specifically ablated in the anterior mesoderm were generated using *Mesp1*-Cre [1], [3], [5], [6], [7]. Embryo heads were collected at E8.5 (5–7 somites) and E9.5 (18–20 somites). Four genotypes were analyzed: CM-CKO (*Twist1*^flox/del^; *Mesp1*^Cre/+^), CM-Het (*Twist1*^flox/wt^; *Mesp1*^Cre/+^), Het (*Twist1*^flox/del^; *Mesp1*^+/+^) and Control (*Twist1*^flox/wt^; *Mesp1*^+/+^). RNA labeling and hybridization was carried out by the Australian Genome Research Facility.

### 2.3 Chromatin Immunoprecipitation

ChIP was carried out using extracts of *TWIST1*-expressing MDCK cells [8]. Cross-linking in 1% formaldehyde, lysis and sonication were carried out as described [1]. Extracts were pre-cleared by incubation with A/G magnetic beads (Dynal) for 3 hrs and incubated with an anti-TWIST1 monoclonal antibody (Abcam ab50887) overnight at 4 °C, before adding blocked beads and subsequent washing steps in RIPA buffer, RIPA/NaCl buffer and LiCL buffer [1]. Sequencing was carried out by the Australian Genome Research Facility.

### 2.4 Data analysis

Raw microarray data were log_2_ transformed, quantile normalized and differential expression analyzed using the Linear Models for Microarray (LIMMA, [9] implementation within Gene Pattern. Differentially expressed genes were filtered on a false discovery rate (FDR) of 0.05.

For ChIP-Seq data, 50 bp reads were trimmed using Cutadapt [10], filtered by quality score and aligned to the CanFam3 dog genome using bowtie2 [11] as described [1]. Peaks were called using MACS2 [12] and IDR analysis performed using an IDR cut-off of 0.05. Peak coordinates from two replicates were merged, using the most extreme start and end positions of the two replicates. The equivalent mouse genome (mm10) peak genomic locations were determined using Liftover (NCBI) annotated using the R library ChipSeeker.
